# School based interventions versus family based interventions in the treatment of childhood obesity- a systematic review

**DOI:** 10.1186/2049-3258-72-3

**Published:** 2014-01-29

**Authors:** Saravana Kumar Kothandan

**Affiliations:** 1 Guys and St Thomas NHS Foundation Trust, Dulwich Hospital, London, England

**Keywords:** Children, Obesity, Family intervention, School intervention, Frameworks, Treatment

## Abstract

**Background:**

The prevalence of childhood obesity, which has seen a rapid increase over the last decade, is now considered a major public health problem. Current treatment options are based on the two important frameworks of school- and family-based interventions; however, most research has yet to compare the two frameworks in the treatment of childhood obesity.

The objective of this review is to compare the effectiveness of school-based intervention with family-based intervention in the treatment of childhood obesity.

**Methods:**

Databases such as Medline, Pub med, CINAHL, and Science Direct were used to execute the search for primary research papers according to inclusion criteria. The review included a randomised controlled trial and quasi-randomised controlled trials based on family- and school-based intervention frameworks on the treatment of childhood obesity.

**Results:**

The review identified 1231 articles of which 13 met the criteria. Out of the thirteen studies, eight were family-based interventions (n = 8) and five were school-based interventions (n = 5) with total participants (n = 2067). The participants were aged between 6 and 17 with the study duration ranging between one month and three years. Family-based interventions demonstrated effectiveness for children under the age of twelve and school-based intervention was most effective for those aged between 12 and 17 with differences for both long-term and short-term results.

**Conclusions:**

The evidence shows that family- and school-based interventions have a considerable effect on treating childhood obesity. However, the effectiveness of the interventional frameworks depends on factors such as age, short- or long-term outcome, and methodological quality of the trials. Further research studies are required to determine the effectiveness of family- and school-based interventions using primary outcomes such as weight, BMI, percentage overweight and waist circumference in addition to the aforementioned factors.

## Background

Childhood obesity is a major public health crisis affecting 155 million school-aged children and young people [1] with a higher prevalence among countries undergoing economic transition [2,3]. Certain regions in the world have a higher prevalence of childhood obesity: more than 30% of children in America and nearly 20% of those in Europe are overweight and obese, with a lower prevalence rate in sub-Saharan African and Asian countries [4,5]. This shows that for the first time in history, global obesity is higher than the 1.02 million people who are hungry and undernourished [6].

Epidemiological data of a sample of German children and adolescents supported that 1.3 million children between the ages of 3 and 17 were obese, of which a proportionate number of children will lead the rest of their lives as obese [7]. This long-term persistence of obesity in childhood will lead to chronic diseases like diabetes, cardiovascular diseases, insulin dependence, arterial hypertension and cancer in adulthood. Moreover, obesity is considered an important nutritional disorder advancing slowly into the developing countries, one, which has an insidious long-term effect [8,9].

Childhood obesity is multifactorial [10] and involves a range of interactions including host (genetic and learned behaviour), agent (energy imbalance) and possible environment (copious intake of food), inactive lifestyle and economic and socio-cultural influences [11]. Apart from the above chronic diseases, the main consequence of childhood obesity is metabolic syndrome characterised by type II diabetes and coronary heart disease [9,12]. For instance, there has been a surge in incidences of type 2 diabetes; in 1994, 5% of children were diagnosed with type 2 diabetes which increased to between 30% and 40% in subsequent years. 85% of those children diagnosed were obese [3,13]. A systematic review by Singh [8] found that the risk of being overweight from childhood to adulthood is at least twice when compared to children of normal weight. Therefore, treatment of childhood obesity is important at the early stage [8].

There are two important factors that could lead to an increase in obesity, namely, genetic predisposition and individual factors. The genetic factor may create a susceptibility to obesity but it cannot be the single most important determinant responsible for obesity in a short span [8,14]. The most important individual factors include nutrition and physical activity. Today’s nutrition typically contains fat and protein, enhanced by sweetened drinks, and a lack of fibre intake with a huge increase in consumption of fast food; sometimes schools have the option to supply fast food to children [9]. Physical factors such as an increase in sedentary lifestyles and the availability and marketing of foods, an increase in the use of computers and television viewing, greater dependence on vehicles for transportation, and decreases in physical activity in schools are considered major determinants of obesity [8].

Apart from the above-mentioned consequences and complications of childhood obesity, findings from studies indicate a significant increase in psychosocial consequences of childhood obesity [15] and in many situations, obese children are stereotyped as unhealthy, academically unsuccessful, socially incompetent, unhygienic and lethargic. Furthermore, obese younger children can develop a negative self-image, which, when reflected in adolescence leads to deteriorating degrees of self-esteem associated with sadness, loneliness, and nervousness and will lead to high-risk behaviour [16].

Considering the various factors, it is not surprising that the treatment of childhood obesity is challenging, despite the increasing number of global research studies and government policies aiming to address the increasing prevalence of childhood obesity [9]. In addition, various government initiatives from increasing physical activity after school and increasing active methods of transport are in the pipeline [17]. Moreover, the International Obesity Taskforce (IOTF) developed in Sydney has set principles to protect children from the commercial promotion of foods and beverages [1].

Ultimately, the management of obesity shares the same basic principles as adults since the primary goal is weight reduction and the maintenance of normal weight [6]. The treatment options for overweight and obese children have two important considerations, namely, pharmacological and non-pharmacological treatment [5]. Pharmacological treatment options range from drugs to surgical intervention. These options support clinicians in the management of obesity and many studies support the clinical management [18]: this is not considered in this review. Although the non-pharmacological management strategies for overweight adults are different from those of children, they share the common principle of increasing physical activity and/or decreasing the intake of high-energy foods and modifying the common shared environment [5,19]. In one of the review by Luttikhuis et al., the investigator identified 64 trials, of which 54 were non-pharmacological lifestyle interventions. Most of the trials had a small sample size and a short-term follow up. In spite of these limitations, the reviewer concluded that family-based intervention with a behaviour program to change the diet, lifestyle, physical activity and thinking patterns proved effective in the treatment of overweight and obesity [5].

The intervention has two important frameworks, family-based intervention and school-based intervention. Many studies demonstrate the importance of quality and quantity of food intake and claim that parents influence the level of activity patterns in schoolchildren [20]. More coordinated assessment of children and their families is needed to establish whether developmental, environmental and psychological factors, which could lead to inactivity and poor eating habits, have on effect on weight gain [21]. Moreover, parents or carers have important and long lasting effects on a child’s eating and physical activity patterns throughout their life [22], and act as a primary mediator for behaviour change [23]. A five- and ten-year study on family behavioural treatment reported that predictors for behavioural change among both children and their parents include self-monitoring and praising the children to influence a change in their behaviour [24]. This study is augmented by the study of Golan and Crow [25] which reported the advantages of using a conventional approach using parents as an exclusive agent. The same study found long-term positive results with 60% of children in the treatment group and 30% in the control group non-obese at the end of the study. One of the critiques about the family-based-intervention is that the amount and kind of interaction between the child and its parents’ behaviour outside the experiment setting is one of the main problems considered. This explains the reasons why the effectiveness of a family weight loss program is difficult to determine [26].

This was enhanced by NHS CRD evaluation through a range of studies to examine the effects of family-based intervention, which focussed on two factors; to assess the parent as the agent and behavioural modification programmes [10]. It has been found that the parent-as-agent group could help to reduce the weight of the children [27].

From a different perspective, schools influence the lives of most children and therefore act as a platform for health education and health promotion regarding diet, physical activity and other healthy behaviour [27]. They also play an active role in encouraging children to adopt and maintain healthy eating habits and increase physical activity (CDC). Veugelers and Fitzgerald [28] examined the efficacy of school-based programs for childhood obesity and concluded that school acts as a platform for children to enhance their future health and wellbeing by eating healthily and encouraging physical activity. Since the framework of school-based intervention may improve and provide social benefits, it will improve the child’s health throughout the critical period of growth and maturation and help them to continue healthy habits throughout their lives [29]. Even though dietary habits, healthy lifestyle education, physical activity and involvement of parents have been accepted as modifiable variables, which are linked to evidence of childhood obesity, a true understanding of all causative factors is imprecise [30]. This made it evident that there is degree of variability prevailing in methodological and theoretical underpinning among school-based programs making the evaluation of the effectiveness of outcomes more complex [29].

Meta-analysis by Suarez et al. [31] found that school-based intervention is effective in decreasing and managing childhood obesity, but not in reducing BMI in intervention groups when compared to control groups. This result contrasts with that of Katz et al. [32] where nutrition and physical activity intervention showed a significant decrease in BMI in the intervention group when compared to the control group. In a review by Sharma [17], the intervention carried out in upper elementary and lower middle schools was most helpful in the treatment and prevention of childhood obesity. Furthermore, systematic reviews by Connelly et al. [33] and Ells et al. [34] showed that the effectiveness of school-based interventions is extremely limited with insufficient quality and outcome, which was recommended through Katz et al. [32]. The reviews also showed that there is only a small number of Randomised controlled Trial’s (RCTs) and only a few on the treatment of childhood obesity [35].

High-quality research evidence focuses on intervention components such as physical activity, lifestyle, drug and surgical intervention for the treatment of childhood obesity [11]. A review by Cochrane collaboration carried out by Luttikhuis and colleagues [5] which focused mainly on components like diet, physical activity and/or lifestyle and social support was found to be effective. In addition, Zenzen and Kridli [29] and Suarez et al. [31] agreed that the above components through a school-based intervention framework were found to be effective in the treatment of childhood obesity. In contrast, Berry [36] reported evidence to support the effectiveness of family-based intervention for childhood obesity. This raised the question as to which one of the frameworks (family-based intervention or school-based intervention) is most effective in treating obesity among children. It became evident through the literature search that previous research had not compared the two frameworks for treatment of childhood obesity. The aim of this review is to provide up-to-date evidence from research studies, which have employed a study design seeking to compare the outcomes of school-based intervention with family-based intervention in the treatment of childhood obesity. It is important to know which strategy is more effective in reducing weight or for maintaining a healthy weight long-term following the treatment [5,37].

## Methods

### Criteria for considering studies for this review

Types of studies:

The study includes data from both short- and long- term randomised control trials (12 weeks – 12 months). The primary studies included in the review focused on the treatment of childhood obesity through two comparing strategies, e.g. school- and family-based interventions. Though randomised control trials contribute least when it comes to how and why some factors affect health and behaviour, they are useful for testing the applied interventions with specific objectives [38].

The included studies reported both short- or long-term follow up and the level of evidence check table was used to assess the level of evidence of RCT and Quasi RCT studies, which were adapted from National institute of clinical excellence (NICE) [39].

### NICE Grading of Evidence and Recommendations [39]

Grading of evidence

Ia: Evidence from systematic review or meta-analysis of randomised controlled trials

Ib: Evidence from at least one randomised controlled trial

IIa: Evidence from at least one well-designed controlled study without randomisation

IIb: Evidence from at least one well-designed quasi-experimental study, such as a cohort study

III: Evidence from well-designed non-experimental descriptive studies, such as comparative studies, correlation studies, case–control studies and case series

IV: Evidence from expert committee reports, opinions and/or clinical experience of respected authorities

Grading of recommendations

A: Directly based on hierarchy I evidence

B: Directly based on hierarchy II evidence or extrapolated from hierarchy I evidence

C: Directly based on hierarchy III evidence or extrapolated from hierarchy I or II evidence

D: Directly based on hierarchy IV evidence or extrapolated from hierarchy I, II or III evidence

Types of participants:

The studies included obese children younger than 18 years at the start of the study of any nationality. The primary studies reflected both the baseline information and post-intervention measurement of the obese children. Children of normal BMI were not considered because the review focus is only on the treatment of childhood obesity through school and family-based intervention.

Intervention:

The interventional components considered for studies includes,

Physical activity

Diet and nutrition

Modifying the diet and exercise behaviour

Health promotion strategies

Or a combination of the above

The above delivered interventions were given through either school or family-based framework

Setting:

Interventions were carried out either at school or in a family setting and depend on the framework used in the particular study.

### Type of comparison

The review compares the school-based intervention with family-based intervention using measurements of the outcomes.

Intervention Personnel

There were no special considerations or restrictions on who delivered the intervention. For example, researcher, PCT professionals, physicians, nutrition/diet professionals, teachers, family members, or health professionals. Nevertheless, the interventions had to be delivered through a family or school setting.

Interventions excluded

Any study that used interventions specifically designed for the prevention of childhood obesity was excluded.

Types of outcome

The studies included in this review reported one or more of the following outcomes including the baseline and post-intervention measurement. Self-reported height and weight measurements were not considered.

Primary Outcomes:

Height and weight

BMI: A validation study by Pietrobelli et al. [40], supports that Body Mass Index can be used to assess body fatness. In addition, this study interprets that BMI should be used cautiously when applying to children in the maturation stage.

BMI z score

Percentage overweight

The studies were included if they reported measurements of body frame (in percentages) and bodyweight (in kilograms) by X-ray absorptiometry along with one of the above. The primary outcome was measured immediately after the completion of the intervention.

Secondary outcomes:

Body fat distribution or waist-hip circumference

Measures such as lipid profile

Behaviour change (activity levels and energy intake)

Cost effectiveness/cost of intervention.

The secondary outcomes were measured during follow up of the studies where the primary outcomes were not significant.

### Search methods for identification of studies

A PICO (Population, Intervention, Comparison and Outcome) framework was applied to identify the studies for this review [41,42]. The search strategy used in this review was developed by Centre for Review and Dissemination [43] to undertake a systematic review of research on effectiveness.

A computerised electronic search was performed to identify relevant articles published between January 2000 and August 2010. The relevant articles were located through a computer-assisted search conducted on Medline-Ovid, Pub med, CINAHL, Science Direct and DARE database. On reading the abstract and full text article, the studies were selected depending on the criteria and graded accordingly. In addition to the database search, reference lists of articles were screened on school-based and family-based interventions to locate more studies to use for the review. Internet Google Scholar and professional networking sites were used to identify vague literature.

### Search methods to identify unpublished and on-going studies

The search for grey literature was carried out to find on-going studies, government reports, working papers, fact sheets, conference proceedings and international papers which are unpublished in databases [44,45]. This review executed a search for grey literature in Open SIGLE (System for Information on Grey Literature in Europe), HSR Proj (Health services research projects in progress), Google Scholar, CRD and CRISP (Computer Retrieval of Information on Scientific Projects) Database.

### Inclusion and exclusion Criteria

Inclusion criteria

The inclusion criteria for this review were randomised controlled trial and quasi- randomised controlled trial in the treatment of childhood obesity. The treatment should include either school- or family-based programs as frameworks that directly or indirectly implement the application of an intervention such as physical activity, behavioural and dietary changes. The research could be written in any language providing it had been peer reviewed and focussed on children below the age of 18 years.

Exclusion criteria

The exclusion criteria for the review includes any intervention programmes specifically designed for the prevention of childhood overweight or obesity, programmes that enrolled children for specific medical problems which may have an impact on interventions for obesity and studies and applied interventions for physical activity, diet or behaviour change without family or school-based frameworks. Community-based programs, literature reviews, qualitative studies and non-randomised trials were also excluded.

### Quality appraisal tool

The methodological quality of randomised controlled trials [RCTs] is commonly used to assess the risk of bias on the trial [46]. This review used the Critical Appraisal Skills Programme (CASP) tool to assess the methodological quality of the included studies adapted from PHRU [47]. This critical appraisal tool is used for analytical evaluations of the quality of research work, particularly the methods applied to avoid biases in the research project [48].

### Data extraction approach

Data extraction for this review involved extracting data from the title, abstract and full text of the primary studies; the amount of information gathered depended directly on the research question. Moreover, a data extraction form, specifically designed to collect the necessary data from the primary research papers, was used. This review adapted the data extraction form from the 2nd edition of Centre for Reviews and Dissemination (CRD) [29,49].

### Data Synthesis

The review used descriptive data synthesis to investigate the effectiveness of the interventions and their differences. Critical analysis of the studies were carried out by highlighting the similarities and differences between the studies to identify the heterogeneity among outcomes, study design, quality and reported effects [49]. The specific objective of this review is to assess the efficacy of school-based intervention versus family-based intervention in the treatment of children with obesity with reported primary outcomes like weight, BMI or BMI *Z* score and percentage overweight.

## Results

**Figure 1:**
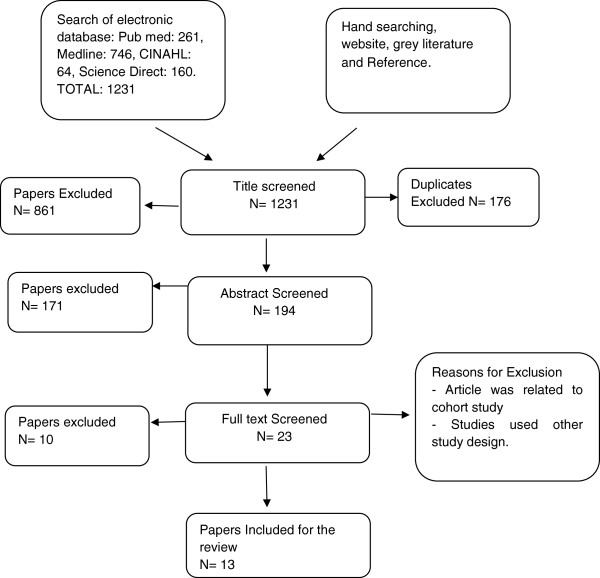
**Flow chart** - **search strategy**.

The initial search yielded 1231 articles from various databases; after screening the titles and abstract of the articles, 23 articles were retrieved and the full text was reviewed. Of the 23 retrieved articles, 13 met the study criteria and were included for this systematic review. All studies included in the review met the NICE Grading of evidence and recommendation level of I b and A (Evidence from at least one randomised controlled trial). Please see the flow chart (Figure 1) and characteristics of included studies in Table 1.

**Table 1:** Characteristics of included studies

**Study**	**Age (years)**	**Population and age at time of outcome assessment**	**Aim/objective**	**Results**
**Family based intervention studies**				
Jiang et al. [67]	<13	Obese children between the grade of 7–9 and their parents of the children.	The focus of the intervention is to evaluate the effectiveness of family based intervention among the school children	- The treatment group showed a statistically significant result in reducing BMI (<0.001) compared to control group
		75 families.		
		participants: n = 68		
		Condition A- treatment group n = 33 (m/f = 20/13)		
		Condition B- control group n = 35 (m/f = 21/14)		
Garipagaoglu et al. [58]	6-14	Obese children of 6–14 years and/ or their parents, BMI value exceeding the 97th percentile for age and gender is taken as inclusion criteria.	The focus of the study is to assess the short and long term effects of family based group treatment in the management of childhood obesity.	The individual treatment group lost more weight than group treatment.
		Self-referred children and their parents.		Statistically significant decrease (BMI, BMI SDS and also in energy intake) is seen among the groups (<0.001)
		Unit of allocation into two groups. participants: 80 (51% male and 49% female)		The decrease in the BMI and BMI SDS is not maintained over the follow up measurement period, however there was decline in BMI seen in individual group
		Condition A Individual treatment (n = 40)		
		Condition B group (n = 40)		
Kalarchian et al. [51]	8-14	Ninety- three Overweight and Obese children between the ages of 8–14 years and their parents were included in the study.	The focus of the study was to evaluate the efficacy of family based weight control in the management of severe childhood obesity.	The planned contrast showed statistically significant differences in percentage overweight in 6 months and small significant result in other medical parameters by 6–12 months. Children who attended
		The participants were recruited through direct mailings, distribution of brochures through local school and community presentations		≥75% of intervention sessions maintained decrease in percentage overweight for 18 months
		Total number of participants: 93		
		a) Condition A- Family Based condition (FB) n = 33		
		b) Condition B- Parent only (PO) n = 34		
		c) Condition C- Wait list control condition (WLC) n = 26		
Golan et al. [59]	8- 11	Children of 6–11 years and their parents	The focus of the study was to evaluate the relative efficacy of treating obesity via a family- based health centred intervention targeting parent only v. parent and obese children.	At the end of the study, treatment was effective among parent only support. Non obese status was also achieved by two children.
		The children were recruited through advertisement in local newspaper.		
		Condition A (parent only) - 14 families with 17 children		
		Condition B (Parent and children) – 18 families with 20 children		
Golley et al. [68]	6- 9	Total 111(64% female) overweight, prepubertal children between 6- 9 years of age and their parents	The focus of the study is to evaluate the effectiveness of parenting skills training as a key strategy for treatment of overweight children.	-All three groups had a statistically significant reduction over 12 months.
		Media publicity and school newsletter.		- In 12 months follow up data shows that BMI z score reduced to 9% in P + DA group, 6% in P group and 5% in WLC group. 45% of children in WLC group increased their BMI z score.
		Condition A - Parenting- skills training with intensive lifestyle education (P + DA) n = 38(13)		- All though there is no statistical significance between the groups, BMI has decreased double the number in 12 months.
		b) Condition B - Parent alone (P) n = 37(13 boys)		
		c) Condition C - Wait listed condition (WLC) n = 36(13 boys)		
**School based intervention studies**				
Sacher et al. [56]	8- 12	Obese children between the ages of 8–12 years and their parents	The focus of the intervention is to evaluate the effectiveness of the Mind, Exercise, Nutrition, do it (MEND) program, a family based community intervention for childhood obesity.	At six months both waist circumference and BMI were highly significantly less in intervention group than control group when adjusted to baseline.
		The participants were recruited from five different UK sites by referral from local health professionals (dieticians, school nurses, and general practitioners) or self-referred.		
		a) Condition A (Intervention group) - 60 allocated (52%)		
		b) Condition B (Wait listed group) – 56 allocated (48%)		
Goldfield et al. [55]	8- 12	Obese children between 8–12 years and their parents.	To evaluate the effectiveness of family based intervention in terms of cost effectiveness intervention.	The result of the study showed that family based behavioural intervention for childhood obesity is cost effective when provided in group format compared to group plus individualised treatment
		Newspaper advertisements and physicians referrals.		
		Condition A (mixed) - 12		
		Condition B ( group only) - 12		
Carrel et al. [52]	12.5 ± 0.5	School going obese children with an age of 12.5 ± 0.5	To determine the effectiveness of school based fitness program to improve fitness, body composition and insulin sensitivity.	There is greater decrease in percentage of body fat in the treatment group compared with control group after end of 9 months intervention
		Recruitment method not mentioned		
		Condition A: 24 in two groups		
		Condition B: 24 in two groups 48% were female		
Francis et al. [60]	Sixth year primary school	Six year primary school children	The focus of the study is to evaluate the effectiveness of short term, school based, and multi-component education intervention on improving the knowledge, attitude and behaviour of primary school children towards obesity treatment.	The participants in the groups had a BMI ≥85th percentile and they showed changes in BMI.
		The district schools were randomly selected by SPSS computer software.		
		Number of participants in each condition		
		a) Condition A n = 56		
		b) Condition B n = 27		
Sahota et al. [53]	7-11	Children between the ages of 7–11 years.	The focus of the study is to assess if a school intervention was effective in reducing risk factor and management of obesity	The program was successful in producing changes in school level.
		Recruitment method not mentioned	The focus of the intervention is to assess o school based intervention in reducing childhood obesity among urban areas in china.	-Only positive outcome increase in consumption vegetable.
		Condition A: n = 314		The study is effective in reducing the childhood obesity among schoolchildren in Beijing.
		Condition B: n = 322		
		Primary school children in grade between 1–4.		
		Five district schools were selected and randomised into intervention and control group.		
		Condition A n = 257		
		Condition B n = 246		
Jiang et al. [67]	1-4 primary year school	Primary school children in grade between 1–4.	The focus of the intervention is to assess o school based intervention in reducing childhood obesity among urban areas in china.	The study is effective in reducing the childhood obesity among schoolchildren in Beijing.
		Five district schools were selected and randomised into intervention and control group.		
		Condition A n = 257		
		Condition B n = 246		
Vissers et al. [57]	17 ± 1.3	Secondary school children with the mean age of 17.5 (±1.3 years)	The focus of the study is to evaluate the effect of multidisciplinary school based intervention for the overweight and obese students attending secondary school.	A school based multi-disciplinary lifestyle intervention is effective and had a promising result to reduce body weight, BMI and improve the aspects of the metabolic syndrome.
		Secondary vocational education schools were contacted to participate in educational project on nutrition, physical activity and health for all students in third grade.		
		Condition A (intervention) n = 37		
		Condition B (control) n = 39		

### Participants

#### Age and gender

All the included studies have obtained data from both males and females. The mean age of participants in the groups was six years old; six studies used participants older than six years [50-54], four studies [50,51,55,56] used participants above the age of eight years, one study by Carrel et al. [52] used participants of 12 ± 0.5 years and a study by Vissers et al. [57] used participants of 17 ± 1.3 years.

### Interventions

Eight trials [58,59] focussed on family-based interventions and the remaining five trials [52-54,57] focussed on school-based interventions with one or a combination of physical activity, diet and nutrition and modifying the diet and exercise behaviour components. Six studies [50,51,55,56,59,67] used all three components in family-based interventions and two studies [57,67] from school-based interventions used all three components. One study [58] from the family-based intervention group and two family-based intervention studies [52,60] utilised physical activity and diet as a components of the intervention. Aside from that, two studies [53,68] used a lifestyle change approach instead of the above-mentioned three approaches. Please refer to Table 2.

**Table 2:** Interventions used in included studies

**Study ID**	**Behavioural**	**Physical activity**	**Diet**	**Others**
Jiang et al. [67]	X	X	X	_
Garipagaoglu et al. [58]	X	_	_	sedentary life style
Janicki et al. [50]	X	X	X	_
Kalarchian et al. [51]	X	X	X	Setting goals
Golan et al. [59]	X	X	X	Life style
Golley et al. [68]	_	_	_	Life style parenting skills
Sacher et al. [56]	X	_	X	Life style program
Goldfield et al. [55]	X	X	X	Stimulus control Reinforcement, self-monitor
Carrel et al. [52]	_	X	X	Fitness/ Lifestyle
Francis et al. [60]	_	X	X	Activities increasing the interest
Sahota et al. [53]	_	_	_	Life style change
Jiang et al. [61]	X	X	X	_
Vissers et al. [57]	X	X	X	Goals

(X) Intervention used (_) Intervention not used.

### Outcomes

#### Weight

Out of thirteen studies included in this review, seven studies [51,55,56,58,59,67,68] from the family-based intervention group and four studies [53,57,60,61] from the school-based intervention group reported weight as an outcome measure. Seven studies [51,55,56,58,59,67,68] reported weight loss in the family-based intervention group and one study from each group [50,52] did not include weight as an outcome measure. Please refer to Table 3.

**Table 3:** Outcome measure used in the included studies

**Study**	**Weight**	**BMI**	**BMI z score**	**Percentage overweight**
Family Based Intervention				
Jiang et al. [67]	X	X	_	_
Garipagaoglu et al. [58]	X	X	_	_
Janicki et al. [50]	_	_	X	_
Kalarchian et al. [51]	X	X	_	X
Golan et al. [59]	X	X	X	X
Golley et al. [68]	X	X	X	_
Sacher et al. [56]	X	X	X	_
Goldfield et al. [55]	X	X	_	X
School based intervention				
Carrel et al. [52]	_	X	_	X
Francis et al. [60]	X	X	_	_
Sahota et al. [53]	X	X	_	_
Jiang et al. [61]	X	X	_	X
Vissers et al. [57]	X	X	_	X

(X) Out measure used (_) Outcome measure not used.

#### BMI and BMI Z score

Twelve studies reported BMI as an outcome measure except Janicki et al. [50] who reported BMI *Z* scores as an outcome measure. Four studies from the family-based intervention group [56-58,60] and two studies from school-based intervention [57,60] showed a significant decrease in BMI. Four studies [50,51,59,68] reported a significant decrease in the BMI Z score.

#### Percentage overweight

Three studies from family-based interventions [51,55,59] and three studies from school-based interventions reported a significant decrease in percentage overweight.

### Quality of reporting

The author assessed the methodological quality of included studies using the CASP tool developed by the public health research unit [47]. All included studies showed some methodological weakness according to the CASP criteria of assessment. Seven studies from the family-based intervention group scored high methodological quality between eight and ten, except a study by Goldfield et al. [55] which reported low methodological quality, whereas on average five school-based intervention studies reported methodological quality between six and seven out of ten. Methodological quality of two studies, one from family-based interventions (Goldfield et al. [55]) and one from school-based interventions (Carrel et al. [52]) scored six out of ten. These two studies scored less points due to a lack of participants, follow up and data collected in the studies. Please refer Tables 4 and 5.

**Table 4:** Methodological quality of included studies

	**CASP questions**	**Jiang et al. **[67]	**Garipagaoglu et al**. [58]	**Janicki et al.**[50]	**Kalarchian et al**. [51]	**Golan et al**. [59]	**Golley et al**. [68]	**Sacher et al**. [56]
1	Did the study ask clearly Focussed Question?	Yes	Yes	Yes	Yes	Yes	Yes	Yes
2	Was this a RCT and it is appropriate so?	Yes	Yes	Yes	Yes	Yes	Yes	Yes
3	Were the participants appropriately allocated to intervention and control group?	Yes	Yes	Yes	Yes	Yes	Yes	Yes
4	Participants, staffs and Intervention Personnel blind to participants?	No	No	No	No	Yes	Yes (single blinded)	Yes
5	Were all the participants who entered the trial are accounted for its conclusion?	Yes	Yes	Yes	Yes	Yes	Yes	Yes
6	Were all the participants in all groups followed up and data collected?	Yes	Yes	Yes	Yes	Yes	Yes	Yes
7	Did the study have enough participants to minimise the chance to play?	Yes	Yes	No	Yes	No	Yes	Yes
8	How the results presented and what are the main results?	Parent, Based, Intervention	Group, Based, Intervention	Parent, Only, Intervention	Family based intervention- short term.	The participant were not enough	SS, P + DA is effective	HighlySS, <0.0001
9	How precise are results?	SS <0.001	SS <0.05	SS Result	SS <0.0001	SS result for parent only group	Yes	Yes
10	Were all the outcomes considered so that result can be applied?	Yes	Yes	Yes	Yes	No	Yes But BMI not reported	yes
	**Total**:	9/ 10	9/ 10	8/ 10	9/ 10	8/ 10	10/ 10	10/ 10

**Note**: Eligibility criteria item doesn’t contribute to total score.

**Table 5:** Methodological quality of included studies

**S. No**.	**CASP questions**	**Goldfield et al**. [55]	**Carrel et al.**[52]	**Francis et al**. [60]	**Sahota et al**. [53]	**Jiang et al**. [61]	**Vissers et al**. [57]
1	Did the study ask clearly Focussed Question?	Yes	Yes	yes	Yes	Yes	Yes
2	Was this a RCT and it is appropriate so?	Yes (Only random allocation)	Yes	Yes	Yes	Yes	Yes
3	Were the participants appropriately allocated to intervention and control group?	Yes	Yes	Yes	Yes	Yes	Yes
4	Participants, staffs and Intervention Personnel blind to participants?	No	No	No	No	No	No
5	Were all the participants who entered the trial are accounted for its conclusion?	Yes	Can’t tell	Yes	Can’t tell	Yes	Can’t tell
6	Were all the participants in all groups followed up and data collected?	No	Can’t tell	Yes	Can’t tell	No	No
7	Did the study have enough participants to minimise the chance to play?	No	No	Yes	Yes	Yes	Yes
8	How are the results presented and what is the main result?	SS in group treatment	SBI effective	SBI effective	SBI effective	SBI effective	SBI effective
9	How precise are results?	< .01	<0.04	SS result	Effective	<0.01	<0.01
10	Were all the outcomes considered so that result can be applied?	No	yes	Can’t tell	Yes	No (some patient lack follow up	yes
	**Total:**	6/ 10	6/ 10	7/ 10	7/ 10	7/ 10	7/ 10

**Note**: Eligibility criteria item doesn’t contribute to total score.

### Allocation

All included studies were sequentially allocated to one of the groups; however, in three studies [52,55,67] the sequence of randomisation was not clear. Out of the thirteen studies included, six studies [50,51,56,58,68] from the family-based intervention group and one study [60] from the school-based intervention group used a randomisation procedure concealed through an opaque envelope and computer generated stratification. The remaining studies [52,53,55,59,67] provided no information about concealment, but participants were allocated randomly into intervention and control groups.

### Incomplete data

Two studies [53,59] in the school-based intervention group had incomplete outcome data and intention to treat analysis was presented in three studies [51,59,68] in the family-based intervention group.

### Other sources of bias

In some family-based interventions studies the sample size varied from n = 24–192 and 48–636, and in school-based intervention studies power calculation was not discussed. A study by Janicki et al. [50] provided $50 for each attendee on assessment in the family-based intervention group and another study by Janicki et al. [50] offered free subscriptions for the fitness centre, which might be risk of bias.

### School-based intervention versus family-based intervention

#### Post treatment weight outcome

Seven studies from family-based and four studies in school-based intervention groups reported statistically significant results with weight as an outcome measure. On the other hand, a study by Sahota et al. [53] from the school-based intervention group showed an insignificant result and a study by Janicki et al. [50] did not report the post intervention weight changes in the result. Studies by [58,67] showed a P < 0.0001.

#### Post treatment BMI outcome

Short term evaluation of family-based intervention studies reported a significant decrease in post intervention value of P < 0.001 and a study by Sacher et al. [56] reported a value of P = .10. A study by Janicki et al. [50] did not report BMI as an outcome, whereas four studies from the school-based intervention group [52,57,60,61] reported a significant reduction in BMI; nevertheless, one by Sahota et al. [53] reported an insignificant decrease.

#### Post treatment BMI Z score

A study by Janicki et al. [50] showed a BMI z score as the only outcome measure which compared three different groups: family-based, parent only and wait listed. The parent only group showed a significant reduction when compared to the waitlist group. However, no significant difference was found between the family-based and parent only groups. Further, a study by Sacher et al. [56], reported P <0.0001 for 6 months and 0.7 during the 12 months follow up. This shows that the reduction in the BMI Z score is not maintained long term, which was not reported in school-based intervention studies.

#### Post treatment percentage overweight

Three studies [51,55,59] from the family-based intervention group and a study [52] from the school-based intervention group reported a significant decrease in percentage overweight. A study by Kalarchian et al. [51] reported a significant reduction by P = 0.0001, .0004 and .02 for six, twelve and eighteen months.

Secondary outcome

#### Grey Literature

In developing a Consensus Statement for childhood obesity, the international assembly presented the evidence, developed recommendations and served a platform, which aimed to offer future remedial actions on international context. Apart from other pharmacological treatment, the consensus statement also emphasised that family-based programmes which include a behavioural programme might be effective in treating obesity among children [69].

#### Excluded studies

Ten studies [4,70-78] were excluded from the review due to the various reasons detailed in Tables 3 and 5.

## Discussion

Thirteen randomised control trials were included in this review, of which eight were based on family-based interventions and five were school-based interventions. The included studies were based only on treatment aspects of childhood obesity by comparing two strategies such as family and school-based interventions. Most of the studies reported positive effects on the treatment of childhood obesity through either family- or school-based intervention frameworks, but the challenge remains as to which of the two interventions is most effective. Whether the question was fully answered or not, the review identified some evidence on the effectiveness of various stages of the interventions in treating childhood obesity.

The included trials for review were heterogeneous and involved children between the ages of 6 and 17 years. Eight studies (n = 8) focused on family-based intervention studies with participants (n = 721) and five (n = 5) for school-based intervention studies with participants (n = 1346). Seven studies in the family-based intervention reported to be effective except for one study by Goldfield et al. [55], which focussed on cost effectiveness of the intervention. The family-based intervention studies reported their effectiveness depending on various factors such as the type of intervention, methodological quality, outcome, follow up and miscellaneous factors such as the setting, intervention personnel and duration of treatment [54].

The interventional components of childhood obesity include behaviour change, diet and physical activity [62]. Family-based interventions utilised all the three components, more specifically dietary behaviour changes, except for one trial by Golley et al. [68], which utilised lifestyle parent skills as a foundation for successful intervention that places a regular, targeted increase in physical activity and targeted reduction in high fat foods. Barlow and Dietz [63] reported that parent-involved programmes had short or long-term beneficial effects on the BMI of participants, which was supported by Jiang et al. [67]. Most of the school-based programmes used physical activity and dietary changes as their intervention, which proved to be effective in the short term [17]. However, a systematic review by Katz et al. [32] found that physical activity and nutrition proved to be effective in decreasing BMI among the intervention group when compared to control. The importance of a combined diet, physical activity and behaviour components were highlighted in many studies [64,65], though was observed in family-based rather than school-based intervention studies. Studies by Golan (1998 and 2006) proved parental involvement as effective components of an intervention to treat childhood obesity, which was also reported in a systematic review by Luttikhuis et al. [5] which showed that parental involvement with children younger than twelve years is more effective than at any other age. In the review, it was shown that parental involvement is more effective between the ages of 6 and 12 years than participants over 12 years. In some school-based intervention, the age group between 4 and 17 years proved to be effective and two more studies [57,60] which included children in the 6th year of primary school and 17 ± 1.3 years of age proved to be effective. This might be because older children may experience more benefits from the school-based intervention, which may be due to fact that they can use the skills taught to then compare themselves to younger children [53,61].

The majority of the studies reported a significant decrease in weight after the treatment, but one study [52] did not report weight as an outcome. Statistically significant results were seen in only two studies [57,67]. Although BMI was the most commonly reported outcome, one study [50] from a family-based intervention trial did not report BMI as an outcome. Statistically non-significant results were reported in three studies, one from family [58] and two from school-based interventions [52,53], which might be due to the duration of each trial. For instance, a study by Carrel et al. [52] had a duration trial of only one month, which was the least duration for the included studies. Apart from that, all included studies lasted between 3 months and 3 years; family-based intervention trials had an average of 3 to 9 months and a one year follow up. Only one study [58] that lasted for three months was too short to offer major results for obesity even though at the end, the study reported a significant difference in the post outcome measurement in treating childhood obesity. It was reported that strategies in the treatment of childhood obesity depend on many other determinants like age of the child, metabolism, needs of child and their stage of maturation, etc.

Few studies used participants as less than n = 24 children in at least one group, [28] this was more seen in family-based studies than school-based studies, which might be due to the intervention setting involved in the trials [66]. For instance, family-based interventions were held in common centres, community venues or university centres or their home and involved children and their parents or carers. In such settings, participants had to make an effort to take part in the study or intervention, whereas in school-based interventions only children took part in their schools, irrespective of their parents.

Studies by Golan et al. [59], Golley et al. [68] and Janicki et al. [50] randomised the participants into family-based, parent and children, parent-only group and wait listed group. Two of the studies [59,68] reported the parent only group as significantly effective compared to the other groups, which was supported by West et al. [79]. Jiang et al. [67] used the participant’s home as the setting for the implementation of intervention, where the dieticians provided the intervention along with their family. This was reinforced by Robertson et al. [78] in his study called ‘families for health’ in the UK. On the contrary, few [29] studies reported the effectiveness of using child and parent in a single group. In contrast, school-based intervention groups with only children were found to be effective, except for a study by Sahota et al. [53].

Two studies [52,53] did not account for the missing data during the analysis, but few performed the analysis based on their intention to treat principles. Intention to treat is most important because many studies dealt with high dropout a rate, which was reported in one-third of some studies. Some studies adjusted their baseline measurement with the post-intervention measurement to take account of the dropout rate. Some studies reported the reasons for dropout as transportation or accessibility to community centres [53], hospitals [56] and university medical centres [51]. Other reasons given for dropouts in school-based intervention trials were due to the transfer of children from one school to another.

The analysis of the efficacy of interventions also carried out by comparing the duration of implementation, which proved that interventions lasting more than 6 months showed a significant change in BMI when compared to a duration of less than 6 months (except Sahota et al. [53] in school-based intervention and Garipagaoglu et al. [58] in family-based intervention). The reason behind the use of longer durations is to allow more time for the participants to lose weight and was predominant in family-based interventions. Additionally, follow up of the study participant plays role in determining the efficacy of treatment [80]. The majority of studies in the family-based intervention group had a follow up period of 10 months to 1 year, but only one study [57] from the school-based intervention group reported a follow up of 6 months. The result from follow up studies showed that the weight loss was not maintained when compared to the intervention during the study. This was reported in a study by Kalarchian and colleagues where the intervention group exhibited a greater increase in percentage overweight and BMI in a one-year follow-up than in the usual care group. A study by Sacher et al. [56] maintained the benefits (decrease in BMI and waist circumference) of the trial up to 9 months out of a one-year follow up. Furthermore, many studies showed improved benefits in their secondary outcomes for blood pressure, heart rate and physical activity levels in the long term.

Appraisal of the studies was carried out through CASP tool, adapted to evaluate the methodological quality of the included randomised controlled trials [47]. The result from the methodological analysis showed some inadequacies, which makes it difficult to illustrate the conclusion of the included studies. The majority of the studies used a minimum of two groups (intervention and control or usual care group) up to a maximum three groups (e.g. family-based, parent and children, parent only or wait-listed group). It was interesting that four studies [52,53,55,67] did not report the randomisation procedure. Out of eight trials in a family-based intervention, five attained a high quality score through the CASP tool. Regarding the number of participants, the study with fewer participants resulted in a low methodological quality, which was seen in Goldfield et al. [55] and compared to Sacher et al. [56] which resulted in a high methodological quality. Most of the school-based intervention trials shared a methodological quality between six and seven. The reason behind the low methodological quality is that, school- based intervention does not report blinding of participants due to most of the intervention held in schools with classroom-based activities and improvement or addition of activities in physical education class, where the blinding of the participants was not feasible [31]. Some of the studies also showed a low attrition rate, which was not accounted for in the conclusion and follow up. In addition, a review by Wilson and colleagues [81] found that a disadvantage in determining the effectiveness of family-based intervention trials is the use of a behavioural component.

Nevertheless, family-based intervention provides several other advantages in reducing obesity, e.g., cost-effectiveness, sharing of experiences, easy implementation of principles to day-to-day life and benefits a greater number of children per professional [51,58,59,67,68]. Likewise, older children may experience more benefits from a school-based intervention, which may be because they can more effectively use the skills taught to them than younger children [53,61]. Moreover, one study reported that five obese children at the end of a trial were categorised as non-obese at the end of the follow-up period [58]. In a few studies [30], a poor compliance rate was reported in both groups, especially among the family-based intervention trials.

The review determined and compared the two strategies, school- and family-based interventions using components of behavioural, diet/nutrition and physical activity. In addition, a comparison was carried out using an appraisal (CASP) tool to check the quality of included studies, which reflected some weaknesses in methodological quality in terms of the number of participants, randomisation procedure and follow up. In previous studies [52,82], the association between physical activity, nutrition, behaviour and obesity was well established. However, there is no conclusive evidence in comparing the strategies because of the heterogeneity that prevails among the included studies to find out which one is effective. Rather, there is evidence suggesting the involvement of parents in a school setting may be beneficial [82,83]. Furthermore, Dietz and Gortmaker [84] concluded that parental involvement is an essential component of any obesity treatment, without which the result will be transitory [36,74].

It has been seen that research in this particular area is difficult to conduct because it involves children, though it is most important in situations where childhood obesity is becoming a global epidemic in terms of nutrition rate [85]. Halting this epidemic is possible through combining treatment strategies along with some preventive initiative. However, heterogeneity data from the trials makes it difficult to conclude that one particular strategy or combination is more effective in the treatment of childhood obesity. Improving our understanding and reporting these findings could inform policy and guide public health efforts in the treatment of childhood obesity.

### Limitations

There are several limitations for the review, which should be acknowledged. The selections of trials were based on eligibility criteria which limited publications to those that met the eligibility criteria between the years 2001 and 2010. Using this CASP checklist to assess quality of reporting is also subjective to bias, as there is a possibility that the user may interpret the criteria differently (in spite of given guidelines). It is unfeasible to ascertain the majority of factors concerning a lack of detailed reporting, which often results from a word limit set by medical journals [82]. It is vital that the researcher reports the standard deviations and confidence intervals in studies, which would make it easier to compute the effect size in determining which intervention is more effective. In addition, the studies using different recruitment methods are an additional limitation for the review. Furthermore, the heterogeneity in the intervention period may confound the intervention results, though it is difficult to avoid this type of heterogeneity.

## Conclusions

A limited conclusion was drawn regarding the effective strategy in the treatment of childhood obesity. One of the important conclusions to be made about the interventions is the theoretical underpinning of each intervention. For instance, family-based interventions used social cognitive theory, the trans-theoretical model, and many other behavioural models in treating children as their parents, whereas school-based interventions lacked theoretical models in their studies. In addition, involving the parents directly in the treatment could yield a more effective outcome. As seen from the included studies, school-based intervention is either ineffective or shows effective short-term results rather than long-term results, whereas family-based behavioural intervention showed a long-term positive outcome for overweight and obese children. In the same way, involving parents directly in school-based intervention adds to the beneficial outcome, instead of considering a separate school and family-based intervention for effective results.

### Implications for practice

It has been seen that limited quantities of data are available to determine the effectiveness of one possible intervention, as both show potential results. Nevertheless, family-based intervention favoured more points in terms of participants, type and duration of intervention and usage of theoretical model for treatment. However, combining both the family- and school-based interventions might be effective for long-term results. Behavioural programs have a promising effect in reducing weight and maintaining a healthy weight in the long term. When it comes to validity, a few studies had a small sample size, greater dropout rate and unadjusted outcome measure, posing the possibility of a small study bias. Moreover, the studies’ findings may be non-generalisable. This review demonstrates that an intervention focusing on short-term strategies (family- or school-based) is not effective in treating obesity. Rather, practitioners need to consider long-term interventional strategies that will have a long-term maintenance effect on obesity.

### Implications for research

Several methodological randomised controlled studies are needed to determine the effectiveness of family- and school-based interventions using primary outcomes such as weight, BMI, percentage overweight and waist circumference. The research studies with the possibility to report some basic primary outcomes would avoid the heterogeneity among the studies because most of the included studies used different outcome measures, which make it difficult to consolidate the effectiveness of any particular strategy. Baranowski and colleagues [86], who reported that the measures of height, weight and BMI are crucial for surveillance, effectiveness and epidemiological research, support this statement. The studies should include experimental research design, which involves physical activity, diet, lifestyle and behaviour in one single strategy. A few of the included studies [52,56,59] had only a few participants, which makes their study uncertain for decision-making. Therefore, studies should use an adequate number of participants, which is more important than any experimental design study and with a reliable outcome measurement. The intervention should be implemented on a long term basis allowing the children to change their behaviour long term instead of the short term approaches implement for one [60] and three [58] months. Long-term follow up measurements of the trials are also lacking in some of the included studies. When it comes to quality of study, only one [68] reported the use of quality assessment; therefore, it is vital to report the quality of the findings using a statement or checklist. This review does not make any definitive implications regarding the components of strategies (physical activity or diet), as more research has already been reported [32].

## Competing interests

The author declares that she has no competing interests.
